# Self-perception of mental health, COVID-19 and associated sociodemographic-contextual factors in Latin America

**DOI:** 10.1590/0102-311XEN157723

**Published:** 2024-03-22

**Authors:** Pablo Roa, Guillermo Rosas, Gloria Isabel Niño-Cruz, Sergio Mauricio Moreno-López, Juliana Mejía-Grueso, Haney Aguirre-Loaiza, Javiera Alarcón-Aguilar, Rodrigo Reis, Adriano Akira Ferreira Hino, Fernando López, Deborah Salvo, Andrea Ramírez-Varela

**Affiliations:** 1 Facultad de Medicina, Universidad de Los Andes, Bogotá, Colombia.; 2 Secretaría de Salud Pública, Cali, Colombia.; 3 Department of Political Science, Washington University in St. Louis, St. Louis, U. S. A.; 4 Universidad Católica de Pereira, Pereira, Colombia.; 5 Escola Superior de Educação Física, Universidade de Pernambuco, Recife, Brasil.; 6 People, Health and Place Research Unit, Washington University in St. Louis, St. Louis, U. S. A.; 7 Prevention Research Center, Washington University in St. Louis, St. Louis, U. S. A.; 8 Programa de Pós-graduação em Tecnologia em Saúde, Pontifícia Universidade Católica do Paraná, Curitiba, Brasil.; 9 Independent researcher, Bogotá, Colombia.; 10 Department of Kinesiology and Health Education, The University of Texas at Austin, Austin, U. S. A.; 11 School of Public Health, UTHealth Science Center at Houston, Houston, U. S. A.; 12 McGovern Medical School, UTHealth Science Center at Houston, Houston, U. S. A.

**Keywords:** Mental Health, Politics, Social Determinants of Health, SARS-CoV-2, Salud Mental, Política, Determinantes Sociales de la Salud, SARS-CoV-2, Saúde Mental, Política, Determinantes Sociais da Saúde, SARS-CoV-2

## Abstract

This study aimed to estimate the prevalence of alterations in self-perceived mental health during the COVID-19 pandemic and their associated factors in four Latin American countries. This is a cross-sectional study based on data collected from adults in 2021 through the *Collaborative Response COVID-19 Survey* by the MacDonnell Academy at Washington University in St. Louis (United States). The sample was composed of 8,125 individuals from Brazil, Colombia, Mexico, and Chile. A generalized linear model for a binary outcome variable with a logistic link and fixed country effects was used. There were 2,336 (28.75%) individuals who considered having suffered alterations in self-perceived mental health. Unemployed individuals (OR = 1.40; 95%CI: 1.24-1.58), those with bad/regular quality of life (OR = 5.03; 95%CI: 4.01-6.31), and those with high socioeconomic status (OR = 1.66; 95%CI: 1.41-1.96) had a higher risk of self-perceived mental health alterations than those with full-time employment, excellent quality, and low socioeconomic status. According to the fixed-effects model, Brazilians living in the country during the pandemic, who disagreed with their government’s decisions (OR = 2.05; 95%CI: 1.74-2.42) and lacked trust in their government (OR = 2.10; 95%CI: 1.74-2.42) had a higher risk of having self-perceived mental health alterations. Nearly 30% of respondents indicated that the COVID-19 pandemic altered their self-perceived mental health. This outcome was associated with political, sociodemographic, and health risk factors. These findings should help policymakers develop post-pandemic community interventions.

## Resumen

Este estudio tuvo como objetivo estimar la prevalencia de alteraciones en la autopercepción de la salud mental durante la pandemia de COVID-19 y sus factores asociados en cuatro países de América Latina. Este es un estudio transversal de datos recopilados de adultos en el 2021 por medio de la investigación *Respuesta Colaborativa a COVID-19* de la Academia McDonnell en la Universidad Washington en St. Louis (Estados Unidos). La muestra estuvo compuesta por 8.125 personas de Brasil, Colombia, México y Chile. El estudio utilizó un modelo lineal generalizado para una variable de desenlace binario con un enlace logístico y efectos fijos por país. En total, 2.336 (28,75%) personas consideraron que habían sufrido alteraciones en la autopercepción de la salud mental. Los desempleados (OR = 1,40; IC95%: 1,24-1,58), aquellos con calidad de vida mala/regular (OR = 5,03; IC95%: 4,01-6,31) y aquellos con alto nivel socioeconómico (OR = 1,66; IC95%: 1,41-1,96) presentaron mayor riesgo de alteraciones en la autopercepción de la salud mental que aquellos con empleo a tiempo completo, excelente calidad y bajo nivel socioeconómico. Según el modelo de efectos fijos, los brasileños que vivían en el país durante la pandemia y que no estuvieron de acuerdo con las decisiones del gobierno (OR = 2,05; IC95%: 1,74-2,42) y no confiaban en su gobierno (OR = 2,10; IC95%: 1,74-2,42) presentaron mayor riesgo de alteraciones en la autopercepción de la salud mental. Casi el 30% de los encuestados indicaron que la pandemia de COVID-19 alteró su autopercepción de la salud mental. Este desenlace se asoció con factores políticos, sociodemográficos y de riesgo a la salud. Estos hallazgos deben ayudar a los formuladores de políticas a desarrollar intervenciones comunitarias pospandémicas.

## Resumo

Este estudo teve como objetivo estimar a prevalência de alterações na autopercepção de saúde mental durante a pandemia de COVID-19 e seus fatores associados em quatro países da América Latina. Este é um estudo transversal de dados coletados de adultos em 2021 por meio da pesquisa *Resposta Colaborativa à COVID-19* da Academia McDonnell na Universidade Washington em St. Louis (Estados Unidos). A amostra foi composta por 8.125 pessoas do Brasil, Colômbia, México e Chile. O estudo utilizou um modelo linear generalizado para uma variável de desfecho binário com uma conexão logística e efeitos fixos do país. No total, 2.336 (28,75%) pessoas consideraram ter sofrido alterações na autopercepção de saúde mental. Os desempregados (OR = 1,40; IC95%: 1,24-1,58), aqueles com qualidade de vida ruim/regular (OR = 5,03; IC95%: 4,01-6,31) e aqueles com alto nível socioeconômico (OR = 1,66; IC95%: 1,41-1,96) apresentaram maior risco de alterações na autopercepção de saúde mental do que aqueles com emprego em tempo integral, excelente qualidade e baixo nível socioeconômico. De acordo com o modelo de efeitos fixos, os brasileiros que viviam no país durante a pandemia, que discordavam das decisões do governo (OR = 2,05; IC95%: 1,74-2,42) e não confiavam em seu governo (OR = 2,10; IC95%: 1,74-2,42) apresentaram maior risco de alterações na autopercepção de saúde mental. Quase 30% dos entrevistados indicaram que a pandemia da COVID-19 alterou sua autopercepção de saúde mental. Esse desfecho estava associado a fatores políticos, sociodemográficos e de risco à saúde. Estes achados devem ajudar os formuladores de políticas a desenvolver intervenções comunitárias pós-pandemia.

## Introduction

The COVID-19 pandemic has had a critical impact on global public health. Governments took several measures to protect their citizens from SARS-CoV-2, such as social isolation and lockdowns, following the World Health Organization (WHO) recommendations. Although these interventions were adopted to preserve the population’s health, these measures disrupted people’s normal behavior and may have had an adverse effect on their mental health ^1^. Previous research showed that, under the strict lockdown, on average 10% of individuals experienced severe psychological distress, while 50% had moderate distress ^2^. These alterations were also impacted by contextual, sociodemographic, and health factors, which influenced changes in people’s mental health following government policies enforcement, such as lockdown during the COVID-19 pandemic ^3^. Accordingly, research conducted during the pandemic revealed that mental health alterations were linked with variables such as social support, financial stability, and the availability of means to meet basic needs ^2^.

The aforementioned findings relate to people’s perceptions of the adopted measures, their trust, and their preference for governments to address the COVID-19 crisis ^4^. Considering the politicization of the public health response to COVID-19, we conjecture that voting for the incumbent and political ideology may be associated with the mental health of citizens, highlighting that political ideology corresponds to political beliefs (i.e., liberal, moderate, or conservative) and that membership in a given political party would thus be associated with preferences for governments ^5^.

Previous research identified these variables as societal circumstances that correlate with alterations in the self-perceived mental health ^2^ ^,^ ^4^ ^,^ ^6^, which is defined as a set of subjective beliefs about ourselves. Recognizing health experiences requires an appreciation of one’s own subjective condition ^7^. Similarly, governments’ preventive measures to face the COVID-19 contagion are linked to their reliability, vote for the incumbent, and respondents’ political ideology. However, there is scant evidence in Latin America on how governments decision-making affected the mental health of the population, when we consider other facilitating factors. As a result, mental health was recognized as an articulated event of COVID-19 in order to inform policymakers about the identified risk factors so that they can carry out specific interventions to protect people’s health. Therefore, this study aims to estimate the prevalence of alterations in self-perceived mental health in individuals aged 18 and older during the COVID-19 pandemic in four Latin American countries in 2020-2021, along with associated factors.

## Materials and methods

### Study design and setting

The research was designed as a cross-sectional and panel study, in which a sample was obtained by periodically surveying a population. Data were acquired in January 2021 through the *Collaborative COVID-19 Response Survey* by the McDonnell Academy at Washington University in St. Louis (St. Louis, United States) as part of the main project *Examining the Influence of Political Ideology in Mitigating COVID-19 in the Americas* ^8^. This online survey was carried out by Netquest (https://www.netquest.com), which built a panel of around 20,000 people in each of the four countries under screening: Brazil, Mexico, Chile, and Colombia. These countries were chosen because they combined Latin America’s largest countries regarding economic growth and population size, a large COVID-19-vulnerable population and, at the time of writing, incumbent governments that ran the gamut from the populist left (Mexico) to the populist right (Brazil) along with governments presided over by established parties with ample governance experience ^9^. Within the contact group, online survey invitations were distributed to reach a representative sample of each country’s population by sex, age, and socioeconomic status.

### Participants and sample size

Nonprobabilistic sampling with an automated quota system was used to collect responses similar to the sociodemographic prevalence in the four Latin American countries ^10^. We targeted individuals aged over 18 years who resided in one of these four Latin American countries during the 2020-2021 COVID-19 period. Observations that lacked complete information for the dependent and independent variables were excluded. There were 169 missing values (2.03%) out of 8,125 total observations in the final sample.

### Survey description

The survey had 38 questions about government policymaking and the political ideology of respondents. COVID-19 transmission and medical care costs, post-pandemic economic growth perceptions, and attitudes toward citizens were also inquired. There were 19 health-related and 26 social/demographic questions ^8^. The questionnaire was self-administered, and designed to last an average of 20 to 30 minutes.

### Dependent variable

The COVID-19 pandemic has had a massive impact on public health, including mental health ^11^, which is defined as the base of emotions, reasoning, interaction, knowledge, resilience, and self-esteem. Therefore, mental health, from a public health perspective, should not be thought of in terms of a psychopathological diagnosis; rather, it is a process that is constructed through humans interrelationships, their emotional well-being, and their context ^12^.

In this context, the variable self-perceived mental health from the COVID-19 items about health-related behaviors was coded as a dichotomous variable (respondents can consider having alterations in their mental health or not) by the question: “In the past two weeks, how often do you feel negative feelings such as blue mood, despair, anxiety, depression?” (The responses to this question were divided between those who replied (often, always) as alterations and those who answered (hardly ever/sometimes) as non-alterations).

### Independent variables: sociodemographic and health factors

Sociodemographic and health factors considered as independent variables were: sex (categorized into female and male); age groups subdivided into life stages: young adults (18 to 26 years), adults (27 to 59 years), and older adults (60 years or more) ^13^; educational level (primary, secondary, and higher education); employment status (full-time, part-time, and unemployed); physical activity (active and inactive); knowledge about COVID-19 (how confident the respondent is about knowing spreading dynamics of COVID-19, categorized into “not sure at all”, “not very sure”, “something sure”, and “very sure”); quality of life in the pandemic (bad/regular, good, and excellent); and socioeconomic status categorized into low (level 1), medium (level 2), and high (level 3), this categorization was adopted because the participating countries classify their socioeconomic levels differently, making it challenging to establish standardized categories among them. Therefore, the participants were queried regarding their preferred socioeconomic status from the options provided.

### Independent variables: contextual factors

The contextual factors considered independent variables were: trust in government, in which participants indicated whether they were neutral, trusted, or did not trust their government; effectiveness of governance strategies, in which participants indicated whether they agreed or disagreed with whether the government had implemented effective COVID-19 control strategies; political ideology, categorized as right, left, and center; and vote for the incumbent, where people indicated whether they had voted for the incumbent president. Furthermore, we included a 4-level country factor (Mexico, Colombia, Chile, and Brazil).

Since the data was collected via a self-administered questionnaire, nine independent variables (educational level, employment status, physical activity, knowledge about COVID-19, quality of life in the pandemic, socioeconomic status, trust in government, effectiveness of governance strategies, political ideology, and vote for the incumbent) and the dependent variable self-perceived mental health were all based on the participants’ subjective impressions.

### Statistical methods

We described country and outcome characteristics using absolute and relative frequencies and proportions. A 0.05 significance level for bivariate analysis using the chi-square (χ^2^) independence test was defined ^14^. For a multivariate statistical analysis, we employed a generalized linear model (GLM) with a dichotomous dependent variable assumed to be Bernoulli distributed and a logistic canonical link to tie its main parameter to predictors. The factors that help to improve the Akaike information criterion (AIC) were integrated after a stepwise selection (p-values < 0.20). The odds ratio (OR) was determined using the exponent of the regression coefficient as a measure of association (Equation 1). We used the deviance hypothesis test and Wald’s tests to determine the final model and the individual significance of each coefficient, respectively ^15^. Furthermore, considering that country-specific contexts may alter respondents’ opinions about trust in government and approval of government interventions ^16^, we employed a fixed effects model. Also, the receiver operating characteristic (ROC) curve was used to evaluate the dependent variable’s sensitivity and specificity. Furthermore, we examined (based on Cook’s distance) potentially leveraged as well as conditionally unusual data points that may be influencing the model intercept and coefficients. All variables tested had at least one significant category that may explain self-perceived mental health variations, according to the Wald’s test. Stepwise variable selection and AIC were also implemented.

The R programming language, version 4.2.1 (http://www.r-project.org), was used to perform statistical analyses. The study obtained ethics approval from the Institutional Review Board of Washington University in St. Louis (approval n. 202007185), and Ethics Committee of the Los Andes University (Universidad de Los Andes, Colombia; approval n. 202009223).

$$log\left(\frac{\hat{p}}{1-\hat{p}}\right)=\beta_{0}+\beta_{1}\chi_{1}+\beta_{2}\chi_{2}+\ldots +\beta_{i}\chi_{i}$$ (Equation 1)

## Results

### Descriptive analysis by country

Regarding sociodemographic and health characteristics, Mexico had the highest percentage of female participants (52.6%), whereas Colombia the highest percentage of males (50.5%). Chile had the highest prevalence among older adults (60 years or more) at 18.8%, Brazil had the highest prevalence among adults (27 to 59 years) at 72.7%, and Mexico had the highest prevalence among young adults (18 to 26 years) at 21.3%. Regarding educational level, Brazil had a greater proportion of participants with primary and secondary education (11.8% and 47.8%, respectively), whereas Colombia had a greater proportion of individuals with higher education (80.6%). Most unemployed individuals were from Chile (50.8%), while part-time workers were a plurality in Mexico (19%) and full-time workers were a plurality in Colombia (43.3%). Most people who assessed the quality of life during the pandemic as excellent and good were from Colombia (16.5% and 55.9%, respectively), whereas most who ranked it as bad/regular were from Chile (53.1%). According to socioeconomic status, most low-level participants were from Chile (48.1%), most middle-level participants were from Brazil (61.7%), and most high-level participants were from Colombia (27.3%) (Table 1).

Chile presented the highest rate of “no confidence” in the government (77.1%), followed by a neutral position and confidence in Mexico (14.4% and 35.4%, respectively). Regarding vote for the incumbent, pluralities in Mexico (28.2%) and Brazil (47.5%) were discordant. Considering political ideology, self-identified right-wing individuals (28.6%) dominated in Brazil, centrists (30%) in Colombia, and the left-wing (33.3%) in Chile (Table 1).

**Table 1:** General descriptive analysis by country of Mexican, Colombian, Chilean, and Brazilian adults in January 2021.

Variables	Mexico (N = 2,049)	Colombia (N = 2,064)	Chile (N = 2,053)	Brazil (N = 1,959)
n (%)	n (%)	n (%)	n (%)
Contextual factors				
Effectiveness of government strategies				
Strongly agree	242 (11.8)	202 (9.8)	121 (5.9)	223 (11.4)
Agree	760 (37.1)	773 (37.5)	533 (26.0)	816 (41.7)
Disagree	663 (32.4)	784 (38.0)	829 (40.4)	593 (30.3)
Strongly disagree	384 (18.7)	305 (14.8)	570 (27.8)	327 (16.7)
Trust in government				
Confidence	726 (35.4)	478 (23.2)	271 (13.2)	511 (26.1)
Neutral	295 (14.4)	284 (13.8)	199 (9.7)	193 (9.9)
No confidence	1,028 (50.2)	1,302 (63.1)	1,583 (77.1)	1,255 (64.1)
Vote for the incumbent *				
Concordance	906 (44.2)	545 (26.4)	866 (42.2)	930 (47.5)
Neutral	366 (17.9)	768 (37.2)	608 (29.6)	406 (20.7)
Discordance	577 (28.2)	560 (27.1)	376 (18.3)	377 (19.2)
Political ideology **				
Left	537 (26.2)	575 (27.9)	684 (33.3)	589 (30.1)
Center	609 (29.7)	627 (30.4)	602 (29.3)	520 (26.5)
Right	406 (19.8)	470 (22.8)	338 (16.5)	560 (28.6)
Sociodemographic and health factors				
Sex				
Female	1,077 (52.6)	1,021 (49.5)	1,078 (52.5)	982 (50.1)
Male	972 (47.4)	1,043 (50.5)	975 (47.5)	977 (49.9)
Age groups (years)				
Young adults (18 to 26)	436 (21.3)	405 (19.6)	296 (14.4)	362 (18.5)
Adults (27 to 59)	1,380 (67.4)	1,454 (70.4)	1,372 (66.8)	1,425 (72.7)
Older adults (60 or more)	233 (11.4)	205 (9.9)	385 (18.8)	172 (8.8)
Educational level ***				
Primary education	30 (1.5)	23 (1.1)	79 (3.8)	232 (11.8)
Secondary education	879 (42.9)	361 (17.5)	627 (30.5)	937 (47.8)
Higher education	1,132 (55.2)	1,664 (80.6)	1,298 (63.2)	776 (39.6)
Employment				
Full-time employability	771 (37.6)	893 (43.3)	742 (36.1)	696 (35.5)
Part-time employability	389 (19.0)	241 (11.7)	268 (13.1)	370 (18.9)
Unemployed	889 (43.4)	930 (45.1)	1,043 (50.8)	893 (45.6)
Physical activity ^#^				
Inactive	505 (24.6)	446 (21.6)	641 (31.2)	595 (30.4)
Active	1,544 (75.4)	1,618 (78.4)	1,412 (68.8)	1,233 (62.9)
Knowledge about COVID-19 (spreading dynamics of COVID-19)				
Very sure	1,136 (55.4)	1,241 (60.1)	1,148 (55.9)	1,139 (58.1)
Something sure	724 (35.3)	679 (32.9)	691 (33.7)	611 (31.2)
Not so sure	157 (7.7)	126 (6.1)	168 (8.2)	153 (7.8)
Not sure at all	32 (1.6)	18 (0.9)	46 (2.2)	56 (2.9)
Social capital and family (infected by COVID-19)				
Very worried	1,806 (88.1)	1,738 (84.2)	1,743 (84.9)	1,366 (69.7)
Worried a little bit	199 (9.7)	258 (12.5)	223 (10.9)	425 (21.7)
Not too worried	40 (2.0)	57 (2.8)	58 (2.8)	114 (5.8)
Not worried at all	4 (0.2)	11 (0.5)	29 (1.4)	54 (2.8)
Pandemic quality of life				
Bad/Regular	896 (43.7)	570 (27.6)	1,091 (53.1)	956 (48.8)
Good	1,000 (48.8)	1,154 (55.9)	860 (41.9)	802 (40.9)
Excellent	153 (7.5)	340 (16.5)	102 (5.0)	201 (10.3)
Socioeconomic status				
High	371 (18.1)	563 (27.3)	267 (13.0)	256 (13.1)
Middle	1,166 (56.9)	849 (41.1)	798 (38.9)	1,209 (61.7)
Low	512 (25.0)	652 (31.6)	988 (48.1)	494 (25.2)

* Missing data for all countries = 840 (10.3%);

** Missing data for all countries = 1,608 (19.8%);

*** Missing data for all countries = 87 (1.1%);

^#^ Missing data for all countries = 131 (1.6%).

### Descriptive analysis by outcome

During the pandemic, 28.8% of the participants reported having alterations in self-perceived mental health and 71.2% reported not having alterations, according to the results (Table 2). Participants’ age ranged from 18 to 82 years (mean ± SD [standard deviation]: 39.9 ± 14 years). The country with the highest proportion of self-perceived mental health alterations was Brazil (32.8%), followed by Chile (26.9%), Colombia (21%), and Mexico (19%). The majority of those who stated no confidence in their government (72.8%) reported the highest proportion of alterations, followed by 16.6% of those who said they had confidence and 10.6% of those who were neutral. According to the vote for the incumbent, “concordance” had the greatest proportion of alterations (38.4%), followed by “neutral position” (25.7%) and “discordance” (25%). Regarding political ideology, people who showed the highest alterations were those who self-identified as the left-wing (34.8%), followed by centrists (28.1%), and the right-wing (18.1%). All the aforementioned variables were statistically significant (p < 0.05 and p < 0.001) (Table 2).

Regarding sociodemographic and health characteristics, unemployed individuals related the greatest alterations in self-perceived mental health (52.5%), followed by full-time employees (32.8%) and part-time employees (14.6%). Most people with alterations in self-perceived mental health ranked the pandemic quality of life index as bad/regular (64%) followed by those who rated it as good (32.2%) and excellent (3.8%). Participants with a middle socioeconomic status had the highest prevalence of alterations (52.6%), followed by those with a low socioeconomic status (29.2%) and those with a high socioeconomic status (18.2%).

The variables education level and knowledge about COVID-19 were not significant (p > 0.05). The relationships between the other variables and the outcome were statistically significant (p < 0.001 and p < 0.01) (Table 2).

**Table 2:** Descriptive analysis and chi-square independence test of self-perceived mental health in Mexican, Colombian, Chilean, and Brazilian adults in January 2021.

Variables	Self-perceived mental health		Overall	p-value
	No alterations	Alterations		
	n (%)	n (%)	n (%)	
Total	5,789 (71.24)	2,336 (28,75)	8,125 (100,00)	
Contextual factors				
Country				< 0.001
Mexico	1,599 (27.6)	450 (19.3)	2,049 (25.2)	
Colombia	1,573 (27.2)	491 (21.0)	2,064 (25.4)	
Chile	1,425 (24.6)	628 (26.9)	2,053 (25.3)	
Brazil	1,192 (20.6)	767 (32.8)	1,959 (24.1)	
Effectiveness of government strategies				< 0.001
Strongly agree	607 (77.0)	181 (23.0)	788 (9.7)	
Agree	2,173 (75.4)	709 (24.6)	2,882 (35.5)	
Disagree	1,980 (69.0)	889 (31.0)	2,869 (35.3)	
Strongly disagree	1,029 (64.9)	557 (35.1)	1,586 (19.5)	
Trust in government				< 0.001
Confidence	1,598 (27.6)	388 (16.6)	1,986 (24.4)	
Neutral	724 (12.5)	247 (10.6)	971 (12.0)	
No confidence	3,467 (59.9)	1,701 (72.8)	5,168 (63.6)	
Vote for the incumbent *				< 0.05
Concordance	2,350 (40.6)	897 (38.4)	3,247 (40.0)	
Neutral	1,544 (26.7)	604 (25.9)	2,148 (26.4)	
Discordance	1,305 (22.5)	585 (25.0)	1,890 (23.3)	
Political ideology **				< 0.001
Left	1,573 (27.2)	812 (34.8)	2,385 (29.4)	
Center	1,701 (29.4)	657 (28.1)	2,358 (29.0)	
Right	1,343 (23.2)	431 (18.5)	1,774 (21.8)	
Sociodemographic and health factors				
Sex				< 0.001
Female	2,766 (47.8)	1,392 (59.6)	4,158 (51.2)	
Male	3,023 (52.2)	944 (40.4)	3967 (48.8)	
Age groups (years)				< 0.001
Young adults (18 to 26)	860 (14.9)	639 (27.4)	1,499 (18.4)	
Adults (27 to 59)	4,107 (70.9)	1,524 (65.2)	5,631 (69.3)	
Older adults (60 or more)	822 (14.2)	173 (7.4)	995 (12.2)	
Educational level ***				0.209
Primary education	255 (4.4)	109 (4.7)	364 (4.5)	
Secondary education	2,018 (34.9)	786 (33.6)	2,804 (34.5)	
Higher education	3,451 (59.6)	1,419 (60.7)	4,870 (59.9)	
Employment				< 0.001
Full-time employability	2,335 (40.3)	767 (32.8)	3,102 (38.2)	
Part-time employability	926 (16.0)	342 (14.6)	1268 (15.6)	
Unemployed	2,528 (43.7)	1,227 (52.5)	3,755 (46.2)	
Physical activity ^#^				< 0.001
Inactive	1,474 (25.5)	713 (30.5)	2,187 (26.9)	
Active	4,215 (72.8)	1,592 (68.2)	5,807 (71.5)	
Knowledge about COVID-19 (spreading dynamics of COVID-19)				0.700
Very sure	3,362 (58.1)	1,302 (55.7)	4,664 (57.4)	
Something sure	1,914 (33.1)	791 (33.9)	2,705 (33.3)	
Not so sure	410 (7.1)	194 (8.3)	604 (7.4)	
Not sure at all	103 (1.8)	49 (2.1)	152 (1.9)	
Social capital and family (infected by COVID-19)				< 0.01
Very worried	4,676 (80.8)	1,977 (84.6)	6,653 (81.9)	
Worried a little bit	843 (14.6)	262 (11.2)	1,105 (13.6)	
Not too worried	191 (3.3)	78 (3.3)	269 (3.3)	
Not worried at all	79 (1.4)	19 (0.8)	98 (1.2)	
Pandemic quality of life				< 0.001
Bad/Regular	2,017 (34.8)	1,496 (64.0)	3,513 (43.2)	
Good	3,064 (52.9)	752 (32.2)	3,816 (47.0)	
Excellent	708 (12.2)	88 (3.8)	796 (9.8)	
Socioeconomic status				< 0.001
High	1,033 (17.8)	424 (18.2)	1,457 (17.9)	
Middle	2,793 (48.2)	1,229 (52.6)	4,022 (49.5)	
Low	1,963 (33.9)	683 (29.2)	2,646 (32.6)	

* Missing data for all countries = 840 (10.3%);

** Missing data for all countries = 1,608 (19.8%);

*** Missing data for all countries = 87 (1.1%);

^#^ Missing data for all countries = 131 (1.6%).

### Factors associated with alterations in self-perceived mental health

People who lived in Brazil during the COVID-19 pandemic (OR = 2.5; 95%CI [95% confidence interval]: 2.18-3.00) were more likely to have alterations in the self-perceived mental health than those who lived in other countries in the Latin America, such as Mexico. People who self-identified as “no confidence” (OR = 1.15; 95%CI: 0.97-1.37), “discordance” (OR = 1.21; 95%CI: 1.04-1.41), and “left-wing” (OR = 1.20; 95%CI: 1.05-1.38) had more chances to have alterations in the self-perceived mental health than neutral and right-wing individuals (Table 3).

Analyses of sociodemographic and health factors revealed that females (OR = 1.61; 95%CI: 1.46-1.78), young adults (OR = 2.75; 95%CI: 2.26-3.35), and physically inactive participants (OR = 1.40; 95%CI: 0.87-1.16) were more likely to have alterations in self-perceived mental health than males, older adults, and physically active participants, respectively. Moreover, unemployed individuals (OR = 1.40; 95%CI: 1.24-1.58), participants who reported bad/regular quality of life during the pandemic (OR = 5.03; 95%CI: 4.01-6.31), and individuals with high socioeconomic status (OR = 1.66; 95%CI: 1.41-1.96) were more likely to have alterations in self-perceived mental health than employed individuals, individuals who reported excellent quality of life, and individuals with low-level socioeconomic status, respectively (Table 3).

**Table 3:** Inferential analysis, general linear model, and Wald’s test of self-perceived mental health with 5,459 total observations excluding missing data in Mexican, Colombian, Chilean, and Brazilian adults in January 2021.

Variables	Model 1 (theoretical)						Model 2 (saturated)		
	Crude OR	95%CI	Pr (> |z|)	Adjusted OR	95%CI	Pr (> |z|)	Adjusted OR	95%CI	Pr (> |z|)
Contextual factors									
Country									
Mexico	1.00			1,00			1.00		
Colombia	1.10	0.90-1.20	< 0.05	1.30	1.1-1.5	< 0.001	1.30	1.10-1.60	< 0.001
Chile	1.60	1.30-1.80	< 0.001	1.40	1.2-1.7	< 0.001	1.50	1.30-1.80	< 0.001
Brazil	2.20	1.90-2.60	< 0.001	2.50	2.1-2.9	< 0.001	2.50	2.10-3.00	< 0.001
Effectiveness of government strategies									
Strongly agree	1.00			1.00			1.00		
Agree	1.09	0.90-1.30	0.2	0.80	0.7-1.09	0.2	0.80	0.70-1.09	0.2
Disagree	1.50	1.20-1.80	0.3	1.10	0.8-1.3	0.3	1.10	0.80-1.30	0.3
Strongly disagree	1.80	1.40-2.20	< 0.05	1.20	1.03-1.6	< 0.05	1.20	1.02-1.60	< 0.05
Trust in government									
Neutral	1.00			1.00			1.00		
Confidence	0.70	0.50-0.80	< 0.05	0.80	0.6-1.0	0.1	0.80	0.60-0.90	0.1
No confidence	1.40	1.20-1.60	< 0.001	1.10	0.9-1.3	< 0.05	1.10	0.90-1.30	< 0.05
Vote for the incumbent									
Neutral	1.00			1.00			1.00		
Concordance	0.90	0.90-1.20	0.07	1.10	0.9-1.3	0.07	1.10	0.90-1.30	0.1
Discordance	1.10	0.80-1.06	< 0.05	1.20	1.03-1.4	< 0.05	1.20	1.04-1.40	< 0.05
Political ideology									
Center	1.00			1.00			1.00		
Right	0.80	0.70-0.90	0.7	1.02	0.8-1.2	0.7	1.03	0.80-1.20	0.6
Left	1.30	1.10-1.50	< 0.001	1.20	1.03-1.4	< 0.05	1.20	1.05-1.30	< 0.01
Sociodemographic and health factors									
Sex									
Male	1.00			1.00			1.00		
Female	1.60	1.40-1.70	< 0.001	1.60	1.5-1.8	< 0.001	1.60	1.40-1.70	< 0.001
Age groups (years)									
Older adults (60 or more)	1.00			1.00			1.00		
Adults (27 to 59)	1.70	1.40-2.10	< 0.001	1.40	1.2-1.7	< 0.001	1.40	1.20-1.70	< 0.001
Young adults (18 to 26)	3.50	2.90-4.20	< 0.001	3.00	2.4-3.8	< 0.001	2.70	2.20-3.30	< 0.001
Educational level									
Secondary education	1.00			1.00			-		
Primary education	1.09	0.80-1.30	0.3	1.10	0.80-1.40	0.3	-	-	-
Higher education	1.05	0.90-1.10	0.4	1.10	1.04-1.30	0.4	-	-	-
Employment									
Full-time employability	1.00			1.00			1.00		
Part-time employability	1.10	0.90-1.30	0.6	0.90	0.80-1.10	0.8	0.90	0.80-1.10	0.7
Unemployed	1.70	1.50-1.90	< 0.001	1.30	1.10-1.40	< 0.001	1.40	1.20-1.50	< 0.001
Physical activity									
Active	1.00			1.00			1.00		
Inactive	1.20	1.10-1.40	< 0.001	1.50	1.10-1.60	< 0.05	1.40	1.20-1.60	< 0.05
Knowledge about COVID-19 (spreading dynamics of COVID-19)									
Not sure at all	1.00			1.00			-		
Not so sure	0.90	0.60-1.40	0.5	0.80	0.50-1.30	0.4	-	-	-
Something sure	0.80	0.60-1.20	0.1	0.70	0.50-1.10	0.1	-	-	-
Very sure	0.80	0.50-1.10	0.2	0.70	0.50-1.10	0.2	-	-	-
Social capital and family (infected by COVID-19)									
Not worried at all	1.00			1.00			1.00		
Not too worried	1.60	0.90-3.05	0.2	1.04	0.60-1.80	0.2	1.01	0.50-1.80	0.2
Worried a little bit	1.20	0.70-2.20	0.8	1.40	0.80-2.70	0.8	1.40	0.70-2.60	0.9
Very worried	1.70	1.08-2.90	< 0.05	1.50	0.90-2.70	< 0.05	1.40	0.80-2.60	< 0.05
Pandemic quality of life									
Excellent	1.00			1.00			1.00		
Good	1.90	1.50-2.50	< 0.05	2.04	1.60-2.60	< 0.001	1.90	1.50-2.40	< 0.001
Bad/Regular	5.90	4.70-7.50	< 0.001	5.60	4.40-7.20	< 0.001	5.03	4.01-6.30	< 0.001
Socioeconomic status									
Low	1.00			1.00			1.00		
Middle	1.20	1.10-1.40	< 0.05	1.30	1.10-1.50	< 0.001	1.30	1.10-1.50	< 0.001
High	1.17	1.02-1.30	< 0.001	1.50	1.30-1.80	< 0.001	1.60	1.40-1.90	< 0.001
AIC	8,570.4						8,564.2		

95%CI: 95% confidence interval; AIC: Akaike information criterion; OR: odds ratio.

As previously indicated, living in Brazil during the pandemic was associated with the highest likelihood of self-perceived mental health alterations compared to living in Mexico. But it is important to look at the effect by country, considering factors like affinity vote for the incumbent and trust in the government. This is because these two factors may be related to the political situation in each country, depending on government’s attempts to mitigate COVID-19.

When considering the interactions between country and vote for the incumbent, on the one hand, and country and trust in government, on the other regression model 3 (Table 4) revealed a statistically significant association between Brazil and vote for the incumbent (discordance) and Brazil and trust in government (no confidence) (p < 0.001). As a result, those who lived in Brazil during the pandemic and had discordance agree with (OR = 2.05; 95%CI: 1.74-2.42) did not trust their government (OR = 2.10; 95%CI: 1.74-2.42) were more likely to have self-perceived mental health alterations than those who had a neutral position and lived in other Latin American countries.

**Table 4:** Fixed effects model including interactions of country and vote for the incumbent and country and trust in government based on self-perceived mental health in Mexican, Colombian, Chilean, and Brazilian adults. January 2021.

Variables	Model 3 (interactions)		
	Adjusted OR	95%CI	Pr(> |z|)
Country*Vote for the incumbent			< 0.001
Country (Brazil)*Vote for the incumbent (discordance)	2.05	1.30-3.10	< 0.001
Country (Chile)*Vote for the incumbent (discordance)	0.90	0.50-1.30	0.6
Country (Colombia)*Vote for the incumbent (discordance)	1.10	0.70-1.70	0.4
Country (Brazil)*Vote for the incumbent (concordance)	1.60	1.10-2.40	< 0.05
Country (Chile)*Vote for the incumbent (concordance)	1.10	0.80-1.60	0.3
Country (Colombia)*Vote for the incumbent (concordance)	1.01	0.60-1.40	0.8
Country*Trust in government			< 0.001
Country (Brazil)*Trust (confidence)	0.90	0.70-1.10	0.2
Country (Chile)*Trust (confidence)	0.60	0.40-0.80	0.3
Country (Colombia)*Trust (confidence)	0.50	0.30-0.60	0.3
Country (Brazil)*Trust (no confidence)	2.10	1.70-2.40	< 0.001
Country (Chile)*Trust (no confidence)	1.30	1.10-1.50	< 0.05
Country (Colombia)*Trust (no confidence)	1.20	0.80-1.70	< 0.05

95%CI: 95% confidence interval; OR: odds ratio.

Note: the asterisk symbol (*) refers to interactions between variables.

Note: country: Mexico (level reference); vote for the incumbent: neutral (reference level); trust in government: neutral (reference level).

**Figure 1:**
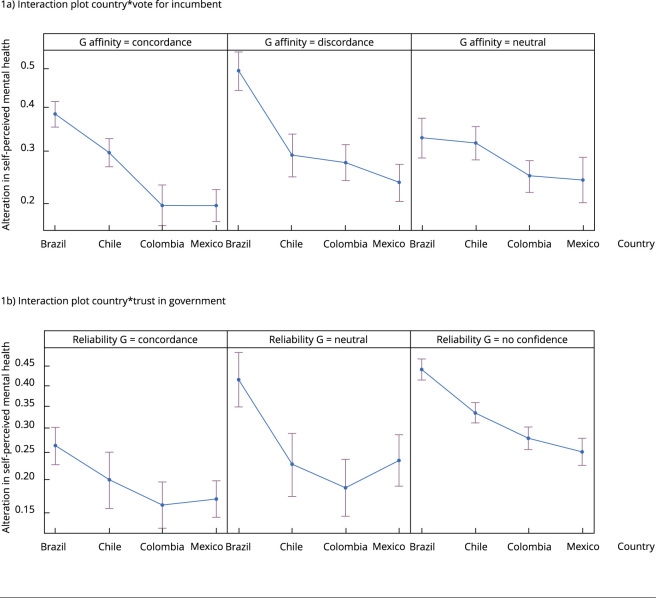
Interaction effect plot: self-perceived mental health in people aged over 18 years by country, vote for the incumbent and trust in government in Mexico, Colombia, Chile, and Brazil, January 2021.

The probability of self-perceived mental health alterations by country was assessed using predictor effects provided by the Effect package of the R program, which yielded graphical summaries fitted with linear predictors (Figure 1), as well as a GLM ^17^. We found that, among the four countries, Brazil’s population has the largest likelihood of self-perceived mental health alterations among those who were discordant (49%) and did not trust their government (44%).

Multivariate model diagnosis showed that the selected model was statistically equivalent to the saturated model (p = 0.812), that the model was significant according to the global likelihood-ratio (p < 0.01) so that at least one coefficient had a linear relationship with the logit of the outcome, and the Hosmer-Lemeshow test confirmed model fit (p = 0.08). According to the ROC curve, self-perceived mental health-affected participants had 72.4% sensitivity and 63.3% specificity.

Finally, 47 data points with a leverage effect were detected from offset residuals greater than four, and Cook’s distance found no extreme data. We continued using model 2 because the significance of the estimated coefficients did not change (Equation 2).

$$log\left(\frac{\hat{p}}{1-\hat{p}}\right)=\beta_{0}+\beta_{1}Co+\beta_{2}EGS+\beta_{3}RG+\beta_{4}AG2+\beta_{5}PA2+\beta_{6}S+\beta_{7}AG+\beta_{9}E+\beta_{12}SC+\beta_{13}Ql+\beta_{14}SEE$$ (Equation 2)

## Discussion

This is the first study in Latin America to examine the prevalence of changes in self-perceived mental health related to the political context of COVID-19 and associated sociodemographic and health factors in Mexico, Colombia, Chile, and Brazil, with the primary objective of obtaining a proportion of 28.8% in these countries.

Multiple research projects investigating politics during pandemics have discovered a significant relation between relevant health-related occurrences and the implementation of containment measures. In this case, our research found that respondents were more likely to report changes in self-perceived mental health if they lacked confidence and disagreed with government policy. According to studies conducted in Latin America ^6^ ^,^ ^11^, agreement with preventive measures and trust in government were linked to the distribution of social resources during lockdowns; consequently, the number of people with mental health issues increased due to difficulties in obtaining government aid, resulting in increased levels of stress and anxiety among vulnerable populations. The inequities in the allocation of social resources not only affected people’s capacity to comply with suggested preventive measures but also intensified preexisting discrepancies. The difficulties in obtaining government assistance further highlighted the systemic obstacles encountered by communities. The mental health consequences of the pandemic went beyond the acute health crisis, highlighting the importance of inclusive policies and focused treatments to address both the virus and its associated social and mental health impacts in this intricate network of components. In the wake of lockdowns, it is crucial for society to prioritize resilience and provide fair access to resources in order to effectively respond to future public health issues.

People with a left-wing orientation were more likely to report having altered self-perceived mental health in each of the four countries studied. However, this conclusion for a country like Mexico, in which the government was left-wing throughout the COVID-19 outbreak, may be inaccurate. Voting for the incumbent was used to identify countries with a greater likelihood of self-perceived mental health alterations in discordant individuals. Some researchers concluded that left-wing political identification was statistically associated with psychological stress during the COVID-19 pandemic. Nevertheless, it is crucial to acknowledge the intricate and diverse characteristics of political connections and their influence on mental well-being. Although voting for the incumbent was used as an indicator of self-perceived mental health changes, the connection between political ideology and psychological well-being is complex. Research has emphasized that political affiliation was not the only factor influencing stress levels during the pandemic, but it was also influenced variables such as socioeconomic status, access to healthcare, and individual’s coping strategies. Furthermore, the link between identifying as left-wing and experiencing psychological stress highlights the complex relationship between political discussions and mental health results, emphasizing the need for a more thorough investigation into the underlying mechanisms. In the face of global crises and political polarization, it is crucial to take a comprehensive approach to mental health research, that is, considering the various factors that influence individuals’ well-being within their political and social environments ^18^. These studies did not suggest that left-wing and government-discordant individuals face greater risks, but they do establish the relationship between vote for the incumbent, political ideology, and mental health in the COVID-19 context.

The third key finding is related to affinity, which is a construction of relationships between personal interests and government policies, so it is a sum of trust and agreeing with government strategies ^5^ ^,^ ^6^. According to previous analyses of trust in government and vote for the incumbent as well as the fixed effects model, people who had no confidence in their government and lived in Brazil during the COVID-19 pandemic had a higher chance of self-perceived mental health alterations than people in other countries in the region.

We identified the concept of social action as a set of policies that governments use to communicate with citizens to create health experiences ^17^. Governments, policymakers, and political leaders are responsible for ensuring collective and individual health. However, by social action, people became aware that COVID-19 pandemic policies could have negatively affected population health, transcending social unrest and the impact of the alterations in self-perceived mental health. Also, some authors considered that the difference between national and local strategies to deal with the COVID-19 pandemic could hurt people’s trust in the government, which could lead to future worries and changes in the mental health of the population. This is known as “punt politics” ^19^ ^,^ ^20^.

The fourth significant finding of this study is the identification of sociodemographic and health factors associated with an increased risk of self-perceived mental health alterations. During the pandemic, the people who suffered the most impact on their mental health were those who had difficulties keeping or finding employment. This outlook about the lack of employability opportunities leads to the difficulty of having an income to meet financial needs. During the pandemic progression, those who were struggling to maintain or obtain employability were disproportionately impacted in terms of their mental well-being. The ambiguity regarding employability security and financial instability engendered a widespread feeling of apprehension and strain. The connection between challenges in finding employment and mental health issues is complex, since the failure to attain a consistent source of income not only endangers one’s financial security but also affects general contentment and self-worth. The widespread nature of these challenges emphasizes the need for comprehensive support systems that tackle both economic and mental health issues ^21^. However, physical activity was identified as a protective variable due to its ability to improve mental health by reducing symptoms like those seen in the study of anxiety, stress, or depression and improving overall emotional well-being. In addition to its physiological advantages, exercise enhances emotional well-being through endorphin release, cultivation of discipline, and enhancement of self-esteem. As societies tackle mental health issues, it is crucial to prioritize regular physical activity, acknowledging the interdependence of physical and mental well-being ^2^.

Finally, the most important findings concern socioeconomic status and quality of life. These are both multidimensional concepts that depend on factors such as life expectancy, income, culture, access to material goods, etc. According to previous research, people in the lowest quintiles of poverty were more likely to develop anxiety and depression due to lower quality of life ^22^. Notably, the countries examined in this study are confronted with significant socioeconomic disparities, exacerbating the influence of poverty on mental well-being ^21^, which may have altered health-related social gradients and risks. Bad/regular quality of life and high socioeconomic status were more likely to alter their perceptions of their mental health. Our results - differing from the literature - suggest that this association could be explained by high-status people not being able to maintain their standard of living due to the pandemic, affecting their quality of life and causing anxiety and depression. Some authors argue that COVID-19 pandemic produced an economic contraction that affected entire societies. Such broad perspective illuminates the complexity among socioeconomic classes, underlining that the economic difficulties arising from the worldwide health crisis have affected even individuals in historically privileged positions. The notion that COVID-19 pandemic triggered a pervasive recession that impacted entire societies is consistent with the broader ramifications of the epidemic on various aspects of life. The economic consequences go beyond individual experiences, infiltrating cultural frameworks and adding to a shared feeling of uncertainty and stress ^23^.

### Strengths and limitations

The participants’ mental health in this study could have been affected before or after the onset of the COVID-19 pandemic. This is a limitation of observational study designs due to the fact that they do not track participants over time to estimate the incidence of an outcome as a result of exposure to a specific setting. However, at the same time, it is a strength within the social determinants of health framework, which suggests a multidimensional view of mental health that helps in identifying people at risk based on correlated characteristics ^5^. It is important to note that self-reported data can be affected by participant’s bias and overestimation of the true frequencies of health conditions ^24^. This study detected self-perceived mental health alterations with a 72.4% sensitivity, demonstrating that personal perception is an early indicator of mental health changes.

## Conclusions

Three out of 10 survey participants reported sadness, anxiety, or depression during the COVID-19 pandemic. Our study explored how different correlates, especially those of a political nature, exacerbated self-perceived mental health outcomes during some of the toughest months of the COVID-19 pandemic in four Latin American countries. Respondents who reported not voting for the incumbent president and those that had no trust in their government were also more likely to report changes to self-perceived mental health. Similarly, implicit risk factors such as unemployment, bad/regular quality of life, or socioeconomic level are also important correlates of changes to self-perceived mental health in the four studied countries. In a post-pandemic scenario, our findings could let policymakers create community interventions that include professionals and community mental health actors to reduce self-perceived mental health changes.
